# Poor performance of two rapid immunochromatographic assays for anti-Japanese encephalitis virus immunoglobulin M detection in cerebrospinal fluid and serum from patients with suspected Japanese encephalitis virus infection in Laos

**DOI:** 10.1093/trstmh/trx067

**Published:** 2017-11-29

**Authors:** Onanong Sengvilaipaseuth, Josée Castonguay-Vanier, Anisone Chanthongthip, Ooyanong Phonemixay, Soulignasack Thongpaseuth, Manivanh Vongsouvath, Paul N Newton, Tehmina Bharucha, Audrey Dubot-Pérès

**Affiliations:** 1 Lao-Oxford-Mahosot Hospital-Wellcome Trust Research Unit (LOMWRU), Microbiology Laboratory, Mahosot Hospital, Vientiane, Lao People’s Democratic Republic; 2 Centre for Tropical Medicine and Global Health, Nuffield Department of Medicine, University of Oxford, Churchill Hospital, Oxford, UK; 3 Division of Infection and Immunity, University College London, London, UK; 4 UMR ‘Émergence des Pathologies Virales’ (EPV), Aix-Marseille Université, IRD 190, Inserm 1207, EHESP, IHU Méditerranée Infection, Marseille, France

**Keywords:** Central nervous system infection, Immunoassays, *Japanese encephalitis virus*, Laos, Rapid diagnostic tests, South-East Asia

## Abstract

**Background:**

*Japanese encephalitis virus* (JEV) is a leading identified cause of encephalitis in Asia, often occurring in rural areas with poor access to laboratory diagnostics. We evaluated two rapid diagnostic tests (RDTs) for anti-JEV immunoglobulin M (IgM) detection.

**Methods:**

Consecutive cerebrospinal fluid and serum from 388 patients (704 samples) with suspected JEV infections admitted to six hospitals in Laos were tested with one of two SD-Bioline anti-JEV IgM RDTs and the World Health Organization standard anti-JEV IgM enzyme-linked immunosorbent assay (ELISA; Panbio Japanese Encephalitis–Dengue IgM Combo ELISA.

**Results and Conclusions:**

The performance of both RDTs showed strikingly low sensitivity in comparison to anti-JEV IgM antibody capture ELISA (2.1–51.4%), suggesting low sensitivity of the RDTs. We highlight the fundamental prerequisite to validate RDTs prior to use to ensure that they meet standards for testing.

## Introduction

*Japanese encephalitis virus* (JEV) is a leading cause of encephalitis in Asia, with an estimated 69 000 cases per year, 30–50% associated neurological sequelae and 30% mortality.^1^ It is also an important cause of undifferentiated fever.^2^ Patients live predominantly in poor, rural communities, often with limited access to laboratory diagnostics. Our knowledge of the true extent of the burden of JEV is limited by the accuracy and accessibility of currently available diagnostic tests.^3,4^

Evidence for the utility of point-of-care diagnostic tests in resource-constrained settings is steadily accumulating.^5,6^ They are, indeed, rapid, easy to use, do not require specific technical knowledge or equipment and have demonstrable accuracy for some pathogens, such as *Plasmodium* spp. and *Dengue virus* (DENV). However, the tests need to be rigorously evaluated, including field testing, before implementation for diagnosis.

Rapid tests for detecting JEV infection have been reported, although none appear to be in routine use.^7–10^ Three of the tests are not convenient for rural diagnostics, requiring overnight incubations and multiple washing steps with various reagents, and one has only been optimized and validated for use in swine. We performed an evaluation of two Standard Diagnostics’ Bioline (SD-Bioline) anti-JEV immunoglobulin M (IgM) rapid diagnostic tests (RDTs) in a study of consecutive cerebrospinal fluid (CSF) and serum of patients with suspected JEV infections in Lao People’s Democratic Republic (Laos).

## Materials and methods

### Ethical statement

This evaluation was part of studies of the causes of central nervous system (CNS) infections and undifferentiated fever in Laos. Written informed consent was obtained from all recruited patients or responsible guardians. Ethical clearance was granted by the Ethical Review Committee of the Faculty of Medical Sciences, National University of Laos and the Oxford University Tropical Research Ethics Committee, Oxford, UK.

### Patients

Patients of any age admitted to four hospitals in Vientiane (Mahosot, Sethatirat, Mother and Child, and Friendship) with suspected CNS infection, without contraindications for lumbar puncture (LP), and who gave informed consent were included.^11^ No formal definition for CNS infection was used for recruitment, which was at the discretion of the responsible physician, reflecting local clinical practice. We also included in- and outpatients from two hospitals outside Vientiane (Luang Namtha in northwest Laos and Salavan in southern Laos), ages 5–49 years, with fever of <9 days without obvious causes, admission tympanic temperature of ≥38°C, including suspected CNS infection, who gave written informed consent.^12^ Blood and/or CSF were collected at admission and a follow-up blood sample was taken at discharge. Serum and CSF were aliquoted and frozen at −80°C.

### JEV RDT

SD-Bioline JE IgG/IgM RDT (catalogue number 48FK20; Standard Diagnostics, Kyonggi-do, South Korea) and SD-Bioline JEV IgM (catalogue number 48FK10; Standard Diagnostics) are immunochromatographic assays for the qualitative detection of anti-JEV IgM antibody from serum, plasma and whole blood. The first group of patients, with undifferentiated fever or suspected CNS infections, were tested using the IgG/IgM RDT. After this test was discontinued, a second group of patients with suspected CNS infection were tested using the IgM RDT. Serum and CSF were tested in this study as per the manufacturer’s instructions. A 5 μl sample was applied to the small ‘S’ well on the cassette, then four drops of diluent (provided with the kit) were applied on the larger round well. The IgG/IgM RDT was tested on one set of samples read by four readers. The IgM RDT was then tested on a second set of samples from different patients by two readers. A valid test showed a pink line ‘C’ in the result window and a positive result (detection of anti-JEV IgM) with a pink line ‘M’ for the IgG/IgM test or line ‘T’ for the IgM test. Results were reported as positive or negative. We did not analyse the anti-JEV IgG RDT results or perform anti-JEV IgG enzyme-linked immunosorbent assays (ELISAs). The RDTs were tested on acute and convalescent serum and CSF, although CSF testing is not included as part of the instructions from the manufacturer.

### Panbio ELISA

Detection of anti-JEV IgM using anti-JEV IgM antibody capture (MAC)-ELISA is the World Health Organization recommended test for diagnosing JEV infection. The Japanese Encephalitis-Dengue IgM Combo ELISA kit (catalogue number E-JED01C; Panbio ELISA; Inverness Medical Innovations, Brisbane, QLD, Australia, formerly Panbio Ltd) is a commercial anti-JEV MAC-ELISA kit that is combined with an anti-DENV IgM test to differentiate IgM against DENV and JEV. The test was performed according to the manufacturer’s instructions, and reported as anti-JEV or anti-DENV IgM positive, equivocal or negative.^13^ Anti-DENV IgM positive or equivocal and anti-JEV IgM equivocal were interpreted as anti-JEV IgM negative.

### Statistical analysis

Anti-JEV MAC-ELISA (Panbio ELISA) was used as a reference standard (‘the best available method for establishing the presence or absence of the target condition’) for the calculation of RDT sensitivity and specificity.^14^ ELISA and RDT results were analysed in a 2×2 table, with sensitivity and specificity (95% confidence intervals [CIs]) calculated using STATA 13.1 (StataCorp, College Station, TX, USA).^15^

## Results and discussion

Two hundred patients (337 samples), 54 with undifferentiated fever and 146 with CNS presentations, were tested by SD-Bioline JE IgG/IgM RDT and 188 CNS presentations (367 samples) were tested by SD-Bioline JEV IgM RDT. A summary of the patient characteristics is presented in Table 1.

**Table 1.** Summary of patients included in the study

	Study group 1 (SD-Bioline JEV IgG/IgM RDT)			Study group 2 (SD-Bioline JEV IgM RDT)			
Admitting hospital	Mahosot	Luang Namtha	Salavan	Mahosot	Sethatirat	Mother and Child	Friendship
Number of patients	140	39	21	143	7	18	20
Age (median, interquartile range)	26 (17–40)	18 (10–26)	18 (13–28)	28 (16–40)	35 (26–48)	6 (3–8)	30 (20–39)
Sex, n (% female)	19 (13.6)	19 (48.8)	7 (33.3)	60 (42.0)	3 (43.0)	5 (28.0)	3 (15.0)
Clinical presentation*, n (% suspected CNS infection)	140 (100)	5 (12.8)	1 (4.8)	143 (100)	7 (100)	18 (100)	20 (100)

*The clinical inclusion criterion for study group 1 was either suspected central nervous system (CNS) infection or undifferentiated fever and for study group 2 was only suspected CNS infection.

Both RDTs demonstrated very low sensitivity with the Panbio ELISA: 51.4% (95% CI 34.0–68.6) for suspected CNS infections and 16.0% (95% CI 4.5–36.1) for undifferentiated fever with the SD-Bioline JE IgG/IgM RDT and 2.1% (95% CI 0.1–11.1) for suspected CNS infections with the SD-Bioline JEV IgM RDT, suggesting low sensitivity of the assay. See Table 2 for individual analyses of CSF, acute and convalescent serum for the two RDTs for patients with suspected CNS infections and undifferentiated fever. The Panbio ELISA detected anti-JEV IgM in 28% of patients, consistent with published data.^1^

**Table 2.** 2×2 Tables comparing anti-JEV IgM detection by Panbio ELISA kit and SD-Bioline JEV RDT results for serum, CSF, all samples and all patients

SD-Bioline JE IgG/IgM RDT^a^										SD-Bioline JEV IgM RDT^b^				
Suspected CNS infection					Undifferentiated fever					Suspected CNS infection				
CSF		Panbio ELISA^c^			CSF		Panbio ELISA^c^			CSF		Panbio ELISA^c^		
		POS	NEG	Total			POS	NEG	Total			POS	NEG	Total
RDT	POS	12	1	13	RDT	POS	0	0	0	RDT	POS	0	0	0
	NEG	17	29	46		NEG	0	0	0		NEG	40	140	180
	Total	29	30	59		Total	0	0	0		Total	40	140	180
Sensitivity (95% CI): 41.4% (23.5–61.0)					Sensitivity (95% CI): —					Sensitivity (95% CI): 0.0% (0.0–8.8)				
Specificity (95% CI): 96.7% (82.8–99.9)					Specificity (95% CI): —					Specificity (95% CI): 100% (97.4–100)				
Acute serum		Panbio ELISA			Acute serum		Panbio ELISA			Acute serum		Panbio ELISA		
		POS	NEG	Total			POS	NEG	Total			POS	NEG	Total
RDT	POS	4	0	4	RDT	POS	3	0	3	RDT	POS	0	0	0
	NEG	4	83	87		NEG	15	31	46		NEG	40	123	163
	Total	8	83	91		Total	18	31	49		Total	40	123	163
Sensitivity (95% CI): 17.7% (15.7–84.3)					Sensitivity (95% CI): 16.7% (3.6–41.4)					Sensitivity (95% CI): 0.0% (0.0–8.8)				
Specificity (95% CI): 100% (95.7–100)					Specificity (95% CI): 100% (88.8–100)					Specificity (95% CI): 100% (97.1–100)				
Convalescent serum		Panbio ELISA			Convalescent serum		Panbio ELISA			Convalescent serum		Panbio ELISA		
		POS	NEG	Total			POS	NEG	Total			POS	NEG	Total
RDT	POS	9	0	9	RDT	POS	4	0	4	RDT	POS	1	0	1
	NEG	3	77	80		NEG	19	26	45		NEG	6	17	23
	Total	12	77	89		Total	23	26	49		Total	7	17	24
Sensitivity (95% CI): 75.0% (42.8–94.5)					Sensitivity (95% CI): 17.4% (5.0–38.8)					Sensitivity (95% CI): 14.3% (0.4–57.9)				
Specificity (95% CI): 100% (95.3–100)					Specificity (95% CI): 100 (86.8–100)					Specificity (95% CI): 100% (80.5–100)				
All patients		Panbio ELISA			All patients		Panbio ELISA			All patients		Panbio ELISA		
		POS	NEG	Total			POS	NEG	Total			POS	NEG	Total
RDT	POS	18	1	19	RDT	POS	4	0	4	RDT	POS	1	0	1
	NEG	17	110	127		NEG	21	29	50		NEG	47	140	187
	Total	35	111	146		Total	25	29	54		Total	48	140	188
Sensitivity (95% CI): 51.4% (34.0–68.6)					Sensitivity (95% CI): 16.0% (4.5–36.1)					Sensitivity (95% CI): 2.1% (0.1–11.1)				
Specificity (95% CI): 99.1% (95.1–100)					Specificity (95% CI): 100% (88.1–100)					Specificity (95% CI): 100% (97.4–100)				

^a^SD-Bioline JE IgG/IgM test and ^b^SD-Bioline JEV IgM test (RDT), both produced by Standard Diagnostics, Kyonggi-do, South Korea. Positive (POS)=‘M’ (IgM) for the IgG/IgM assay and ‘T’ (test) for the IgM assay, and ‘C’ (control) lines visible; negative (NEG)=only ‘C’ line visible. IgG results are not presented. The study was performed in two phases: (1) SD-Bioline JEV IgG/IgM RDT tested on 200 patients with assays read by four independent investigators (interreader agreement 99.4% [95% CI 97.9–99.9]) and (2) SD-Bioline JEV IgM RDT tested on 188 patients with assays read by two independent investigators (interreader agreement 100% [95% CI 99.0–100]).

^c^JEV-Dengue IgM Combo ELISA kit (Panbio ELISA), Inverness Medical Innovations, Brisbane, Australia (formerly Panbio Ltd). Positive (POS)=JEV IgM positive, negative (NEG)=JEV IgM negative or equivocal, with cut-offs calculated according to the manufacturer’s instructions.

For the patient data, positive (POS) refers to CSF and/or serum positive for anti-JEV IgM and negative (NEG) is all available samples negative.

RDTs are currently being used for the diagnosis of a wide range of infectious diseases. SD-Bioline RDT assays for dengue, trypanosomiasis and HIV have been shown to have good diagnostic accuracy.^16–19^ We found that these two JEV RDTs had low sensitivity with reference to ELISA. Similar results for some malaria RDTs have been reported.^20^ The JEV RDTs were supplied directly from the manufacturer and used within the expiry date. Sensitivity was higher for convalescent as compared with acute serum, and this is likely due to higher JEV IgM titres arising over time.^21^ The manufacturer’s in-house assessment reported in the product leaflet for the IgM RDT involved 375 samples with a reported sensitivity and specificity >90% compared with the SD-Bioline anti-JEV MAC-ELISA (catalogue number 48EK10; Standard Diagnostics). In our study, the specificity was consistently high (>90%). Interreader agreement between the two readers was 99.4% (95% CI 97.9–99.9) for the IgG/IgM RDT and 100% (95% CI 99.0–100) for the IgM RDT. Discrepant results were excluded from the analysis. The positive control strip was valid in 700/704 tests (99.4%) and the four invalid tests were repeated successfully.

Limitations of the study include that the evaluation was only in one country and that the sera from the undifferentiated fever study^11^ were not consecutive. We have reported sensitivity and specificity in comparison with ELISA, therefore interpretation of these results should be considered with caution. Indeed, the field is further complicated by very little evidence on the comparability between the many different anti-JEV and anti-DENV antibody ELISAs.^13^ We used a different ELISA system than that used by the manufacturer as stated in their product leaflet. Additionally, cross-reactivity with other antigenically similar flaviviruses were not evaluated, such as confirming ELISA results by the plaque reduction neutralization test (PRNT)—the gold standard—and testing RDT against anti-DENV IgM/IgG-positive serum.^22^

The potential use of RDTs in the diagnosis and surveillance of neglected tropical diseases such as JEV is still emerging. Our results highlight the fundamental prerequisite to validate RDTs prior to use to ensure that they meet standards for testing.^16–19^ There is the potential for cross-reactivity with other flaviviruses circulating in a region, and results must be interpreted with this in mind. Simple, reliable and cost-effective rapid tests are still urgently needed for the diagnosis of JEV infection.
